# Brain-model neural similarity reveals abstractive summarization performance

**DOI:** 10.1038/s41598-024-84530-w

**Published:** 2025-01-02

**Authors:** Zhejun Zhang, Shaoting Guo, Wenqing Zhou, Yingying Luo, Yingqi Zhu, Lin Zhang, Lei Li

**Affiliations:** 1 School of Artificial Intelligence, Beijing University of Posts and Telecommunications, Beijing, 100876 China; 2 Beijing Big Data Center, Beijing, 100101 China

**Keywords:** Deep language models, Representational similarity analysis, Abstractive summarization, Electroencephalography, Neural correlates, Language, Biomedical engineering, Computer science

## Abstract

Deep language models (DLMs) have exhibited remarkable language understanding and generation capabilities, prompting researchers to explore the similarities between their internal mechanisms and human language cognitive processing. This study investigated the representational similarity (RS) between the abstractive summarization (ABS) models and the human brain and its correlation to the performance of ABS tasks. Specifically, representational similarity analysis (RSA) was used to measure the similarity between the representational patterns (RPs) of the BART, PEGASUS, and T5 models’ hidden layers and the human brain’s language RPs under different spatiotemporal conditions. Layer-wise ablation manipulation, including attention ablation and noise addition was employed to examine the hidden layers’ effect on model performance. The results demonstrate that as the depth of hidden layers increases, the models’ text encoding becomes increasingly similar to the human brain’s language RPs. Manipulating deeper layers leads to more substantial decline in summarization performance compared to shallower layers, highlighting the crucial role of deeper layers in integrating essential information. Notably, the study confirms the hypothesis that the hidden layers exhibiting higher similarity to human brain activity play a more critical role in model performance, with their correlations reaching statistical significance even after controlling for perplexity. These findings deepen our understanding of the cognitive mechanisms underlying language representations in DLMs and their neural correlates, potentially providing insights for optimizing and improving language models by aligning them with the human brain’s language-processing mechanisms.

## Introduction

A key goal of Natural Language Processing (NLP) is to enable machines to understand and generate language like humans. Recent advances in Deep Learning (DL) techniques have led to remarkable progress in Deep Language Models (DLMs) across various NLP tasks, with these models demonstrating language understanding and generation capabilities comparable to those of humans^1,2^. This phenomenon has prompted researchers to investigate the similarities and differences between the internal mechanisms of DLMs and human language cognitive processing, attempting to explain the connection between Artificial Intelligence(AI) and biological intelligence in language processing^3–5^. DLMs have provided a new computational framework for studying the cognitive neural mechanisms of human language understanding^5–7^, while drawing on knowledge from cognitive neuroscience has also offered new perspectives for optimizing and improving DLMs^8–10^.

Numerous studies have demonstrated that DLMs exhibit similarities in Language Representation (LR) to the human brain and can, to a certain extent, explain the brain’s activation patterns in response to language^11–13^. Pioneering research has found that Transformer-based DLMs (such as GPT and BERT) can predict functional Magnetic Resonance Imaging (fMRI) and Electrocorticography (ECoG) activation in language-related human brain regions within a noise tolerance range, substantially outperforming traditional Recurrent Neural Network (RNN) models^14^.^15^ discovered that a model’s performance on the next-word prediction task is highly correlated with its ability to explain brain activation. This finding suggests that the human brain may primarily rely on a predictive coding mechanism to understand language, continuously predicting the likely upcoming words during the comprehension process^16,17^. Recent empirical studies have provided further support for this theory. Research^18^ has provided strong evidence for hierarchical predictive processing in natural language understanding. Brain responses to words were found to be modulated by predictions at multiple levels, from phonemes to meaning, supporting theories of hierarchical predictive coding in language processing. Additional investigations demonstrated that enhancing language model activations with long-range predictions improved their mapping to brain responses, revealing a hierarchical organization of language predictions in the cortex. Frontoparietal cortices were shown to predict higher-level, longer-range, and more contextual representations compared to temporal cortices, emphasizing the importance of context in language understanding^19^.Expanding on these findings, researchers have delved deeper into the intrinsic connection between the internal representations of DLMs and human brain language processing. In terms of semantic representation,^5,6,20^ found that the contextual word embeddings of DLMs like GPT-2 can capture the geometric structure of activation in the human brain’s language areas (such as the left Inferior Frontal Gyrus, IFG), outperforming traditional static word embeddings (like GloVe). This result indicates that DLMs have learned context-dependent lexical semantic representations similar to the human brain’s.

Regarding factors influencing similarity,^21^ discovered that the activation similarity between GPT-2 and the human brain in semantic-related brain regions (such as the left temporal lobe) increases with a person’s level of semantic understanding, and this similarity is most evident in the deeper layers of the model^22^. DLMs exhibit hierarchical representation characteristics, with shallow layers of models like BERT encoding surface information, middle layers encoding syntactic features, and deep layers integrating long-distance, high-level global semantics^23^. When using DLMs to predict human brain activation, it was found that different layers of different models have varying predictive abilities for human brain language processing. For example, in GPT-2, the long-distance dependency representations in the middle layers best predict human language understanding, while the short-distance attention in the shallow layers best predicts speech encoding^24^; in BERT, the middle layers perform best in predicting human brain activation^10^. These studies have uncovered the close connection between the internal mechanisms of DLMs and human language understanding, offering valuable insights for further investigating their similarities.

While many current studies focus on investigating the factors contributing to the similarities between DLMs and the human brain, few have explored how the degree of such similarity relates to model performance. Some research has found that the higher the similarity between language models and the human brain, the better their performance on various benchmarks^12^. However, other studies have indicated that this correlation has limited predictive power for model performance. For example,^14^ discovered that a model’s neural fit (brain score) only significantly correlates with its performance on the next-word prediction task. Furthermore, current research on the internal mechanisms of DLMs mainly focuses on elemental semantic composition at the lexical or sentence level. In contrast, exploration of discourse comprehension mechanisms is relatively insufficient^25,26^. Contextual information is crucial for language understanding^27,28^, yet existing studies have not fully considered the impact of context on model LR. The task of Abstractive Summarization (ABS) provides an ideal research scenario for investigating the discourse comprehension mechanisms of DLMs. Automatic text summarization, which extracts critical knowledge from vast amounts of information, alleviates information overload^29^. It has broad application prospects and research value^30,31^. Compared to other NLP tasks, the ABS task places higher demands on a model’s language understanding ability, as it requires deep encoding and integration of the semantics of an entire text. This task primarily employs encoder-decoder architecture models, such as BART^32^, PEGASUS^33^ as well as T5^34,35^, which can be scaled up to Large Language Models (LLMs) with sufficient parameters^36^. The encoder is responsible for hierarchical semantic encoding of the original text, while the decoder generates text summaries based on the encoded information. Considering the encoder’s crucial role in semantic understanding and integration, exploring the neural correlates of the ABS model’s encoder can help reveal the cognitive mechanisms of the human brain in understanding and integrating discourse semantics.

Regarding the ABS task and models, this study raises two pressing questions. First, the roles of different layers of ABS models in discourse semantic understanding and the Representational Patterns (RPs) of syntactic and semantic information remain unclear. Research has found that the human brain processes semantics and syntax with different spatiotemporal characteristics^37^, and the two exhibit partially overlapping distributed representations in the human brain^38,39^. Consequently, we aim to investigate the following question: What roles do the different layers of the ABS model’s encoder play in integrating discourse semantics, and how are grammatical and semantic information represented within these layers? Exploring the model’s RPs can help us understand ABS models’ hierarchical language encoding mechanisms and their neural basis. Second, the correlation between the similarity of ABS models to human brain LRs and their task performance merits further investigation. Language is a unique, high-level cognitive function of humans, and the human brain has evolved highly complex and efficient language processing mechanisms over a long period. Therefore, the closer a DLM is to the human brain in structure and function, the stronger its language understanding and generation capabilities may be. So, for the more complex ABS task, can the degree of similarity between the model and human brain representations also predict the quality of its generated summaries?

Based on the above analysis of the relationship between DLMs and human brain language processing, we hypothesize that, in the context of the ABS task, the hidden layers of the model that exhibit a higher degree of Representational Similarity (RS) to the human brain’s activation patterns may play a more critical role in the model’s performance on this task. To test this hypothesis, we designed an experiment involving human participants and employed two methods: Representational Similarity Analysis (RSA) and layer-wise ablation. This study used the ABS models PEGASUS and BART as the research objects to investigate the similarity between their encoder hidden layer representations and those of the human brain (i.e., neural similarity) and the contribution of each layer to the summarization task. RSA can reveal the similarity of representational structures between systems (such as DLMs and the human brain) by calculating the correlation between their response patterns to the same set of inputs, overcoming differences in representational forms^13,40,41^. Many different stimuli are input into the target system to compute representational structure, i.e., the geometric properties of the representational space. The activity patterns evoked by each stimulus are measured, and the similarity of responses between all stimulus pairs is calculated. This process quantifies the system’s representational structure. Layer-wise ablation, on the other hand, removes the model’s learned attention layer by layer, examining the contribution of each layer to the ABS task by intervening with the model’s internal representations^10,42^.

**Figure 1:**
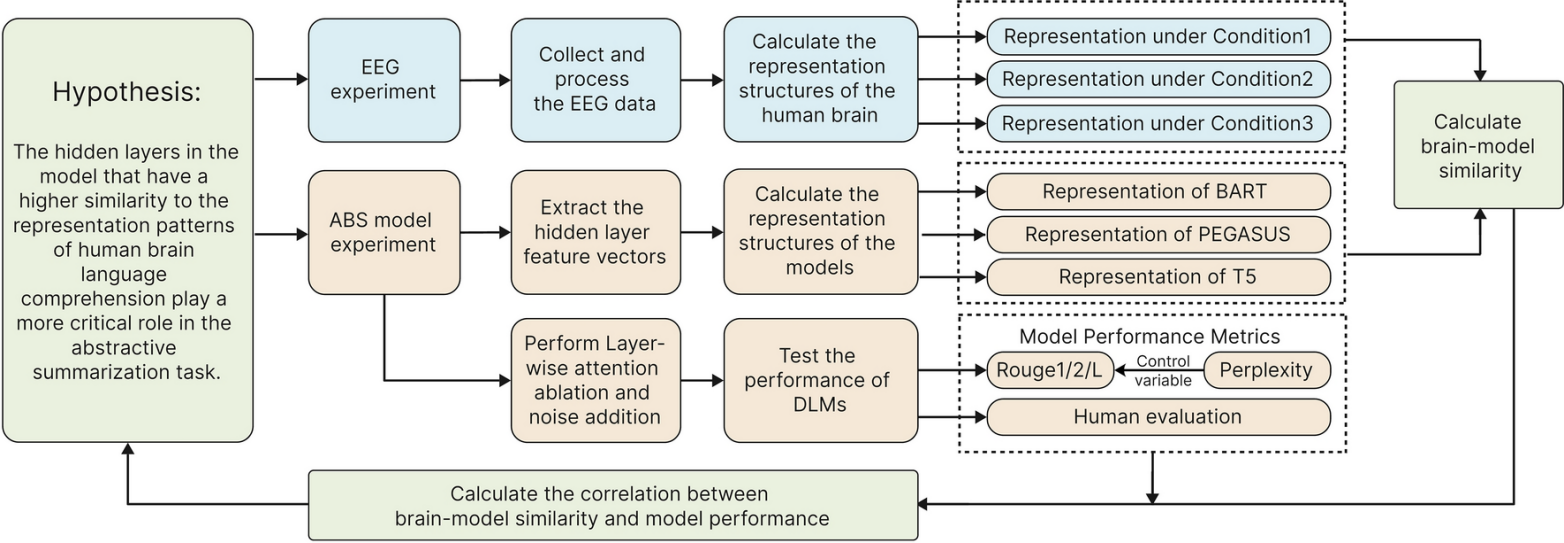
Schematic diagram of the research framework.

Specifically, the N400, a negative-going Event-Related Potential (ERP) component peaking around 400 ms post-stimulus onset, is a neural signature of semantic processing^43^. In contrast, the P600, a positive-going ERP component peaking around 600 ms, is associated with syntactic processing and integration^44^. This study first calculated the similarity between the model’s hidden layer representations and human brain representations under different spatiotemporal conditions. It aimed to reveal the model’s hierarchical language processing mechanism and potential neural correlates. We defined four spatiotemporal conditions: Full time window (using the complete time window and all channels), Early window (using the time window and electrode positions typically associated with the N400 component), Late window (using the time window and electrode positions typically associated with the P600 component) and Baseline (using a pre-stimulus time window and all channels as a control). Then, we performed layer-wise attention ablation and noise addition on each layer, and evaluate the resulting changes in summary quality to investigate the role of each layer in integrating semantics and grasping the text’s overall meaning. Finally, this study connects the RS of each hidden layer of the model to the human brain and its impact on model performance, attempting to test the hypothesis we proposed. Figure 1 illustrates the overall research framework.

Through a series of experiments and analyses, this study makes the following contributions:

1. This study employed RSA to compare LRs in DLMs and the human brain. Results showed increasing similarity with model depth, reaching statistical significance, indicating DLMs’ ability to acquire brain-like LRs. Control experiments revealed the crucial role of model training in this process.
2. The layer manipulation experiment reveals that each encoder layer contributes to the ABS task to a different extent. Ablating the attention mechanism or adding noise in deeper hidden layers leads to a more substantial decline in summarization performance, indicating that deeper layers play a more crucial role in integrating critical information from the original text and understanding its comprehensive theme.
3. This study reveals a significant correlation between hidden layers’ similarity to human brain activity patterns and their impact on model summarization performance. Examining BART, PEGASUS, and T5 models, we found this correlation consistent across diverse spatiotemporal brain activity conditions and layer manipulation methods. The correlation’s persistence after controlling for perplexity further supports our findings’ robustness.

These findings deepen the understanding of the internal working mechanisms of DLMs. They provide valuable insights for optimizing and improving DLMs from a brain-inspired perspective.

## Methods

### EEG signal acquisition and processing

#### Experimental design

Fifteen healthy adult participants were recruited for this study, of which 13 were effective, including nine males and four females, with a mean age of 24.31 ± 2.72 years. All participants were right-handed, had normal or corrected-to-normal vision, and had no neurological or psychiatric disorders history. All participants provided written informed consent prior to their participation in the study.

The BEATs system^45^ was utilized to collect 28-channel EEG signals, as shown in Fig. 3b. The electrodes were placed in an elastic cap according to the standard positions of the international 10-20 system. The 28 electrodes used for recording EEG signals are highlighted in yellow. Based on the research^46^, the electrodes sensitive to the N400 ERP component include Cz, C3, C4, CP1, CP2, Pz, P3, and P4, marked with blue borders in Fig. 3b. The time window sensitive to the N400 component was set to 250-450ms. According to the study^47^, the channels sensitive to the P600 ERP component include C3, Cz, C4, P3, Pz, and P4, marked with red borders in Fig. 3b. The time window sensitive to the P600 component was set to 500-800ms. Throughout the recording process, the sampling rate was set to 250Hz, and the impedance of each electrode was maintained below 10 K$\Omega$ to ensure signal quality.

The experimental program was written using PsychoPy (version 2022.2.5)^48,49^, requiring participants to read a text that appeared word by word continuously. The reading materials were selected from 8 articles in the Factuality Evaluation Benchmark (FRANK) dataset^50^, containing 62 sentences and 1427 words. We present the details of the selected materials in Supplementary Table S1 in the appendix.

**Figure 2:**
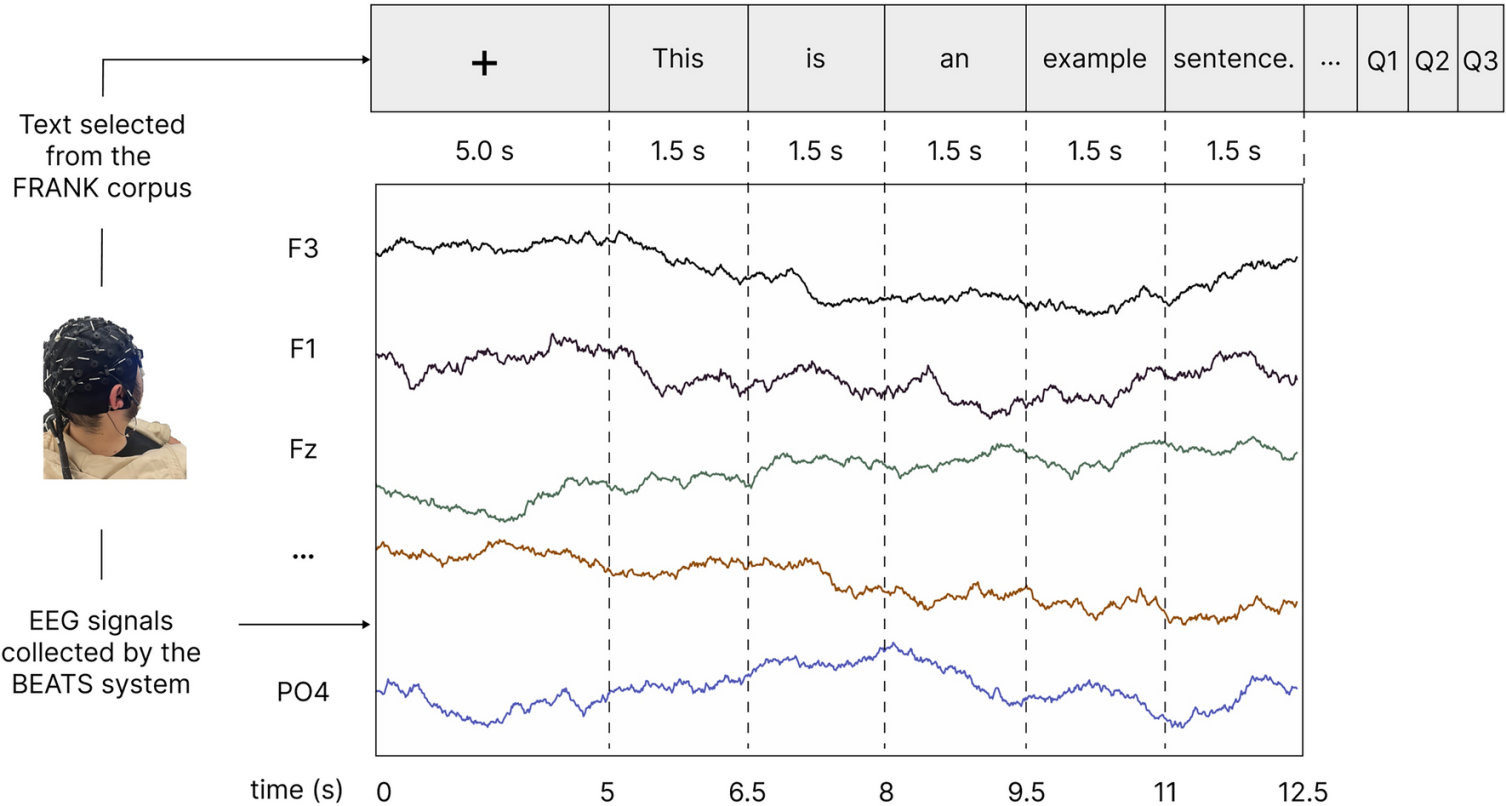
Procedure of the EEG experiment.

Before the experiment, all participants were required to read and voluntarily sign an informed consent form. The experiment was conducted in a soundproof laboratory, with participants seated approximately 60cm from the computer screen. They were required to maintain a horizontal gaze and minimize head movements. The procedure of the EEG signal acquisition experiment is shown in Fig. 2.

First, participants fixated on a central fixation point (a cross) presented on the screen for 5000ms to record baseline EEG signals. Subsequently, participants read articles presented word by word, with each word appearing in the center of the screen for 1500ms, during which the EEG signals were recorded. Finally, after each article was played, participants were required to answer three judgment questions related to the content to ensure they were focused on the reading task. To ensure data quality, only participants who achieved an accuracy rate exceeding 50% for all questions were included in the analysis. After answering the questions, participants had a 2-minute break to avoid potential fatigue effects before starting the experiment with the following article.

#### Data processing

A series of preprocessing steps were performed to remove noise and artifacts from the EEG signals, including filtering, segmentation, baseline correction, artifact removal, and re-referencing. First, the continuous EEG data were bandpass filtered between 0.5Hz and 30Hz to remove low-frequency drift and high-frequency noise. Then, based on the 1459 tokens obtained by PEGASUS’s tokenizer, the continuous data were segmented into discrete time windows. Tokens derived from the same word shared the EEG signals recorded during the presentation of that word. The duration of the EEG signal segment corresponding to each token was 1700ms, including 1500ms of EEG signals during each event (word presentation) and 200ms of EEG signals preceding the event. To avoid signal drift, we performed baseline correction by subtracting the average potential of the 200ms preceding each event from the entire epoch. Next, we identified noisy or faulty channels through visual inspection of the raw EEG data, focusing on channels exhibiting persistent high-frequency noise, flat lines, or excessive artifacts. Then, bad channels were repaired using spherical spline interpolation, and Independent Component Analysis (ICA) was employed to remove artifacts such as eye movements and muscle activity. Finally, a whole-brain re-referencing approach, which means using the mean voltage across all electrodes as the reference, was performed to obtain EEG signals relative to the global average potential. We used Python 3.7.9 and SPSS version 26 for EEG data processing and statistical analysis.

### Experiments on ABS models

This experiment explores the intrinsic mechanisms of discourse comprehension in ABS models. Given the crucial role of the encoder in text comprehension within the model and the focus of EEG experiments primarily on human language comprehension rather than generation, this study centers on analyzing the characteristics of the encoder. The study utilizes three prevalent DLMs that have been fine-tuned for ABS tasks: BART, PEGASUS, and T5^35^. Specifically, the BART-large model is fine-tuned on the CNN/DM dataset^51^, while the PEGASUS-large model on the XSum dataset^52^, and the T5-large model on a combined XSum-CNN/DM dataset. CNN/DM and XSum are benchmark datasets used for training and evaluating ABS models, containing news articles paired with human-written summaries. Each model, featuring distinct architectures and varying numbers of layers, is fine-tuned on different datasets, enhancing the robustness of the experimental findings.

Take the PEGASUS-large model as an instance, the encoder consists of 16 hidden layers, each generating a unique representation of the input discourse text. To analyze the impact of the attention mechanism in different hidden layers of the encoder on the model’s performance, we designed an experiment to remove the attention in each layer individually. This approach eliminates the influence of the learned attention in a specific encoder layer while keeping the parameters of the other layers fixed, allowing us to observe its contribution to the model’s performance. The attention is calculated as shown in Equation 1^53^, where ${d}_k$ is the dimension of the keys, $Q=X{W}^Q$ is the query matrix, $K=X{W}^K$ is the key matrix, and $V=X{W}^V$ is the value matrix.

1 $$\begin{aligned} Attention(Q, K, V) = Softmax \left( \frac{QK^T}{\sqrt{d_k}}\right) V \end{aligned}$$

*X* represents the input to the layer, and ${W}^Q$, ${W}^K$, ${W}^V$ are pre-trained parameter matrices. The uniformization of the attention mechanism is achieved by modifying the matrices ${W}^Q$, ${W}^K$, and ${W}^V$ to ensure that the attention produces equal probabilities for the values in matrix *V*, thereby eliminating the influence of the layer’s attention on matrix *V*^10^. Specifically, the ${W}^Q$ and ${W}^K$ matrices are set to zero matrices, and the ${W}^V$ matrix is set to an identity matrix. This modification allows for removing the learned attention effect of the layer during model propagation, facilitating the analysis of the contribution of the encoder’s layer to the model’s performance. In subsequent sections, we refer to the method of setting attention to a uniform distribution as attention ablation. Additionally, we employ another approach to influence model performance called noise addition. This method involves introducing random perturbations drawn from a Gaussian distribution to the weight matrices of each encoder layer, allowing us to examine the impact of controlled noise on model performance.

To assess the impact of layer manipulation on model performance in the ABS task, we employ two different layer-wise manipulations on the BART, PEGASUS, and T5 models: attention ablation and noise addition. We then generate summaries using these manipulated models on the complete test set. The quality of these summaries, which reflects the models’ performance on the ABS task, is assessed using three variants of Rouge (Recall-Oriented Understudy for Gisting Evaluation): Rouge1, Rouge2, and RougeL. These metrics evaluate the overlap between generated and reference summaries at different levels. Rouge1 measures word-level overlap by calculating the ratio of words in the reference summary that appear in the generated summary. Rouge2 assesses the overlap of consecutive word pairs (bigrams) between the generated and reference summaries. RougeL evaluates the sequence similarity based on the longest common subsequence, capturing the overall structural similarity. This comprehensive evaluation allows us to assess summary quality regarding individual words, phrase structures, and overall content alignment. By applying these metrics to summaries generated after manipulating each encoder layer, we can quantify the impact of attention mechanisms and noise introduction on the models’ ABS capabilities.

### Representational similarity analysis

**Figure 3:**
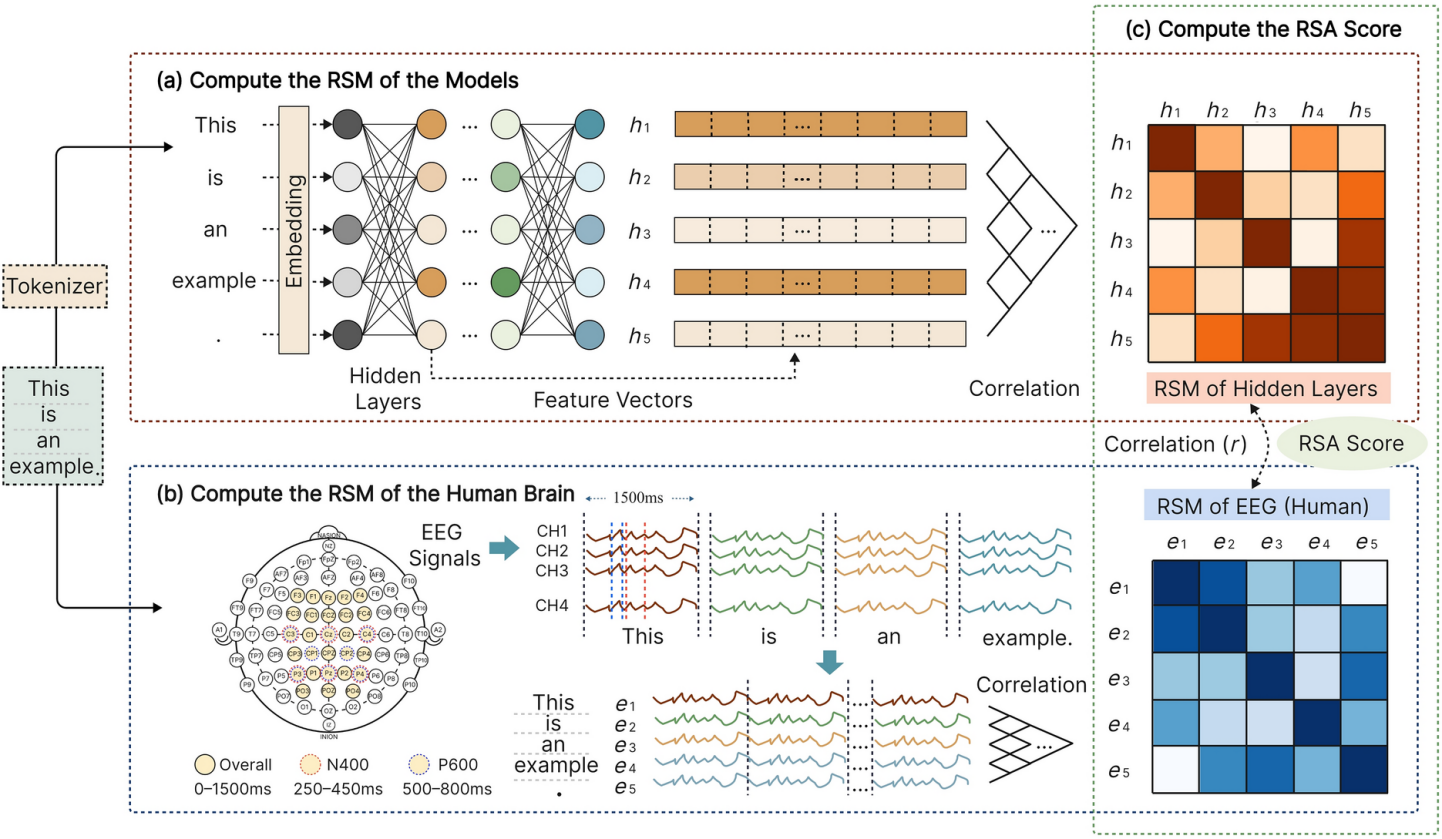
Schematic of representational similarity analysis.

Due to the substantial differences in how language is represented in artificial neural network models and the human brain, directly comparing the correspondence between the two at the word or token level is challenging. Therefore, this study employed the RSA method to compare the activation patterns of models and the human brain in response to input text, i.e., the geometric structure of the representational space. The overall process of RSA is shown in Fig. 3.

For the activation patterns of the model, the cosine similarity was used to calculate the pairwise similarity between the feature vectors corresponding to all tokens in each hidden layer of the encoder, yielding an *n* $\times$ *n* similarity matrix, as shown in Fig. 3a (*n* is the total number of tokens after the tokenizer processes the text; in the example shown, *n* = 5). This matrix characterizes the representational structure of the input text within the model. Specifically, the Representational Similarity Matrix (RSM) ${\textbf{M}}_{ij}^l$ of the hidden layer can be represented as Equation 2, where ${\textbf{h}}_i^l$ represents the feature vector of the *i-th* token in the *l-th* hidden layer, and *n* is the total number of tokens.

2 $$\begin{aligned} {\textbf{S}}_{ij}^l=\frac{{\textbf{h}}_i^l\cdot {\textbf{h}}_j^l}{|{\textbf{h}}_i^l||{\textbf{h}}_j^l|},i,j=1,2,\ldots ,n \end{aligned}$$

Regarding the activation patterns of the human brain, the EEG signals corresponding to each token were similarly represented as a vector and calculated as follows. The EEG experiment recorded the participants’ electrical signals of the human brain while they read all the tokens. After aligning each token with its corresponding word, each token corresponded to the 1500ms EEG signal from the appearance to the disappearance of the word. Given that the sampling rate of the EEG signals was 250Hz, each token had 375 sampling points within the 1500ms time window, accumulating 10500 data points (375 $\times$ 28) across the 28 electrodes. By concatenating the signals from the 28 electrodes, each token corresponded to a 10500-dimensional EEG feature vector. Similar to the model, the similarity of all token pairs in the EEG feature space was computed, and an *n* $\times$ *n* similarity matrix was generated for each participant, as shown in Fig. 3b (*n* = 5 in the example, the EEG signal placed at the right of each word ($e_i$) represents the concatenation of all electrode data for that word), reflecting the representational structure of the human brain during language processing.

We established four spatiotemporal conditions for EEG signals in language comprehension. The condition of Full time window uses the complete time window and all electrodes, providing a comprehensive view of brain activity during language processing. Early window and Late window conditions employ time windows and electrode locations associated with the N400 and P600 components, respectively, capturing brain activity in these specific spatiotemporal windows. Finally, Baseline condition (control group) uses the time window from -200 to 0 ms before stimulus onset and all electrodes as a comparison baseline. Traditional ERP components inspire these conditions; we posit that EEG signals under these conditions may contain information relatively sensitive to human brain semantic (Early window) or syntactic (Late window) processing. However, we do not assume a direct, exclusive mapping between these components and specific linguistic processes. The selection of these conditions primarily aims to observe brain activity patterns during language processing from multiple perspectives, thereby enabling the examination of RS across different spatiotemporal windows. The composition of the EEG vectors under the four conditions is shown in Table 1.

**Table 1:** Composition of the three EEG vector types.

EEG vector	Selected electrodes	Time windows (ms)	Sampling points	Vector dimension
Full time window	All electrodes	0 to 1500	375	10500
Early window	C3, Cz, C4, P3, Pz, P4, CP1, CP2	250 to 450	50	400
Late window	C3, Cz, C4, P3, Pz, P4	500 to 800	75	450
Baseline	All electrodes	− 200 to 0	50	400

The similarity matrix ${\textbf{B}}_{ij}^{k,t}$ of the human brain’s representation of text can be represented as Equation 3, where ${\textbf{e}}_i^{k,t}$ represents the EEG vector recorded for the *k-th* participant while reading the *i-th* token, based on the representation type t, which can be overall representation, semantic representation, or syntactic representation.

3 $$\begin{aligned} {\textbf{B}}_{ij}^{k,t}=\frac{{\textbf{e}}_i^{k,t}\cdot {\textbf{e}}_j^{k,t}}{|{\textbf{e}}_i^{k,t}||{\textbf{e}}_j^{k,t}|},i,j=1,2,\ldots ,n \end{aligned}$$

Using the n$\times$n RSM, this study characterizes the language comprehension process of the model and the human brain in a unified manner. Based on these RSMs, we calculate the correlation between them to investigate the RS between different hidden layers of the model and the brain’s overall, semantic-specific, and syntactic-specific representations. The Spearman rank correlation coefficient was used to calculate the correlation between the RSMs of the model and the human brain, yielding the RSA score:

4 $$\begin{aligned} \rho ^{lk,t}=\frac{\sum _{i=1}^{n(n-1)/2}\ (r_i^l-{{\overline{r}}}^l)(r_i^{k,t}-{{\overline{r}}}^{k,t})}{\sqrt{\sum _{i=1}^{n(n-1)/2}\ (r_i^l-{{\overline{r}}}^l)^2}\sqrt{\sum _{i=1}^{n(n-1)/2}\ (r_i^{k,t}-{{\overline{r}}}^{k,t})^2}} \end{aligned}$$

In Equation 4, $r_i^l$ and $r_i^{k,t}$ represent the ranks of the upper (lower) triangular part (excluding the diagonal) of ${\textbf{S}}_{ij}^l$ and ${\textbf{B}}_{ij}^{k,t}$, respectively, when sorted by similarity values. ${{\overline{r}}}^l$ and ${{\overline{r}}}^{k,t}$ are the corresponding average ranks. Since the similarity matrix is symmetric about the diagonal and the values on the diagonal are always 1, the Spearman rank correlation coefficient calculation is based on the upper triangular part of the matrix, excluding the diagonal elements, as shown in Fig. 3c. Furthermore, this study first calculates the correlation between each participant’s EEG RSM and the RSM of each hidden model layer. Then, it averages the correlations across participants to record the correlation between the representation of that hidden layer and the human brain.

## Experimental results

### RS between human brain and models

**Figure 4:**
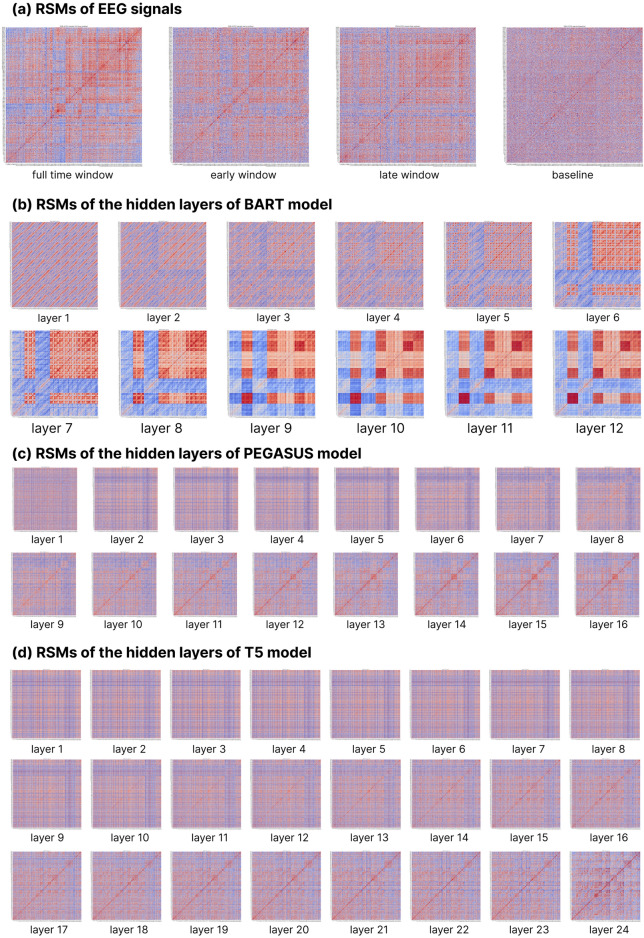
Visualization of RSMs.

To systematically investigate the RPs across the human brain and DLMs, we first conducted comprehensive visualization analyses of their RSMs using rank-based correlation heatmaps. Figure 4 presents these matrices, where color intensity denotes the strength of RS: darker red indicates more substantial similarity and darker blue indicates weaker similarity. We constructed these heatmaps based on rank ordering rather than absolute values to align with our subsequent Spearman rank correlation analyses.

Visual analyses of these matrices revealed distinct architectural features in the representational spaces. A characteristic cross-shaped pattern emerged consistently across the human brain and model RSMs, indicating systematic similarities in specific RPs. In human brain RSMs (excluding baseline conditions), the core of the cross-pattern is localized in the lower-left quadrant of the heatmaps. Notably, BART’s RSMs demonstrated remarkable architectural convergence with human brain RPs, resembling this characteristic lower-left positioning of the cross-pattern core. In contrast, PEGASUS and T5 initially exhibited divergent organizational principles, with their cross-pattern cores predominantly concentrated in the upper-right quadrant. This difference aligns with our later findings that BART achieved higher performance in the RSA.

**Figure 5:**
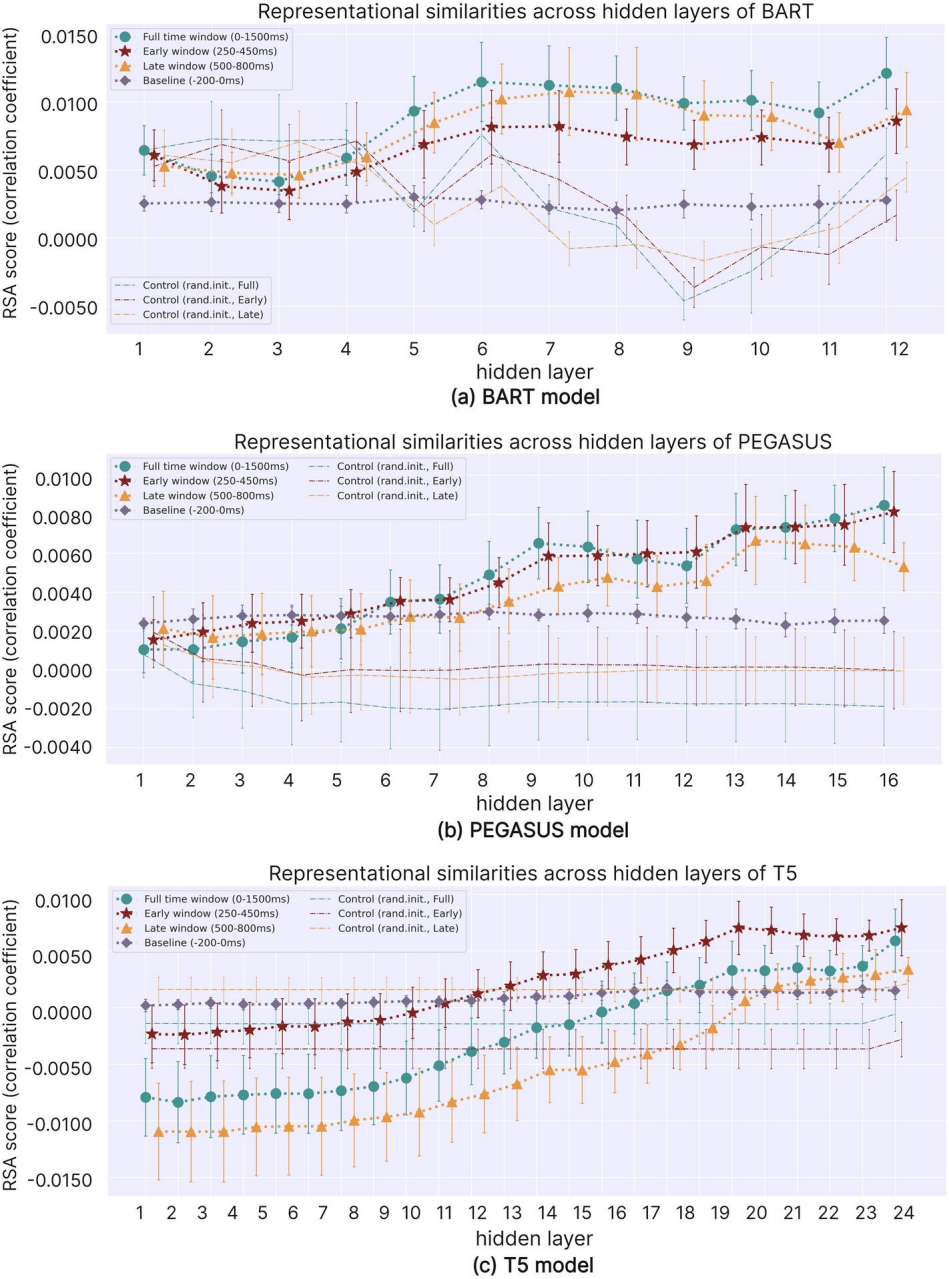
RSA scores between each hidden layer of the DLMs and the human brain.

Figure 5 illustrates the relationship between the degree of RS (RSA scores) of the hidden layers in BART, PEGASUS, and T5 models and human brain activation during language processing. The error bars represent the *standard error* (*SE*), serving as uncertainty estimators and provide a visual representation of data dispersion. For each model, we calculated seven sets of RSA scores: Full time, Early, Late window and Baseline from Table 1, and three control groups for the former three. We randomly initialized the model parameters in the control groups and processed the same text input as the standard ABS models. Then, we calculated the RSA scores between these models and human brain RPs. The control group results, represented by thin lines without markers in Fig. 5, help demonstrate the effect of language model training and fine-tuning on representational capabilities.

While RSA’s effect sizes (correlation coefficients) appear relatively small, we suggest they are meaningful and interpretable for several reasons. First, our visualization analysis of the RSMs reveals systematic similarities in RPs between the human brain and DLM representations, particularly the characteristic cross-shaped structures in blue. These geometric configuration matches substantiate the meaningful relationships underlying the low correlation coefficients. Second, given the scale of our RSMs ($1459\times 1459$), the actual sample size for correlation computation reaches the order of one million, making even small effect sizes statistically reliable at such large scales^54^. Finally, the core strength of RSA lies in capturing the overall geometric structure of representational spaces rather than superficial linear relationships. Therefore, despite modest effect sizes, RSA effectively reveals the fundamental similarities in information encoding patterns between human brains and DLMs.

We employed the Jonckheere–Terpstra test to assess the presence of a statistically significant monotonic trend in the seven sets of RSA scores for each model as the number of layers increased. This statistical analysis was performed using SPSS (version 26), and the results are recorded in Table 2. The sign of *Z* indicates the trend direction, with positive and negative values representing increasing and decreasing trends. The *p*-value < 0.05 indicates statistical significance.

**Table 2:** Jonckheere–Terpstra test results of RSA scores under different conditions.

**RSA conditions**	**BART**		**PEGASUS**		**T5**	
	***Z***	***p***	***Z***	***p***	***Z***	***p***
Full time window	2.843	0.004**	5.694	<0.001***	7.578	<0.001***
Early window	2.858	0.004**	4.837	<0.001***	8.120	<0.001***
Late window	1.643	0.100	3.331	0.001**	8.062	<0.001***
Baseline	− 1.861	0.063	− 0.054	0.957	3.413	0.001**
Control (rand.init., Full)	− 2.975	0.003**	− 0.831	0.406	0.058	0.954
Control (rand.init., Early)	− 3.313	0.001**	− 0.649	0.516	0.026	0.980
Control (rand.init., Late)	− 3.969	<0.001***	− 0.919	0.358	0.030	0.976

*$$p<.05$$; **$$p<.01$$; ***$$p<.001$$.

For the three experimental groups (Full time, Early and Late window), the Jonckheere–Terpstra test revealed that the PEGASUS and T5 models exhibited a statistically significant monotonic increase in RSA scores as the number of layers deepened (*Z*> 0, *p* < 0.05). The BART model showed a statistically significant monotonic increase in RSA scores for Full time and Early window (*Z* > 0, *p* < 0.05), but for Late window, the increasing trend did not reach statistical significance (*Z* = 1.643, *p* = 0.100). Theses findings indicate that the neural networks of DLMs progressively learn LRs that more closely resemble those of the human brain. Through successive layers of abstraction and integration, these deep neural networks encode text in a way that increasingly parallels human cognitive processes. This evolution may be attributed to the models’ shift from extracting superficial lexical features to integrating complex semantic information, aligning more closely with the mechanisms of human language comprehension^23^.

For the four control groups, the Jonckheere–Terpstra test showed no statistically significant monotonic trend in RSA scores as the layers deepened for the BART and PEGASUS models under Baseline. The T5 model exhibited a statistically significant monotonic increase (*Z* = 3.413, *p* = 0.001). Still, Fig. 5 reveals that its RSA scores were close to 0 with minimal fluctuation compared to other groups, indicating no apparent learning effect or similarity to human brain representations despite statistical significance. For the randomly initialized control groups (Control (rand.init., Full time, Early and Late)), the BART model showed a statistically significant monotonic decrease in RSA scores (*Z* < 0, *p* < 0.05), while the PEGASUS and T5 models showed no significant trend. This finding suggests that without specific training, deepening layers in DLMs leads to either decreased (BART) or unchanged (PEGASUS and T5) similarity between the model’s hierarchical representations and human brain representations, underscoring the importance of appropriate training for learning brain-like representations.

We conducted Friedman tests to determine if there were differences among the RSA scores of the three experimental groups (Full time, Early and Late window) for each model. The results revealed statistically significant differences for all models: BART ($\chi ^2(2)$ = 22.17, *p* < 0.001), PEGASUS ($\chi ^2(2)$ = 12.88, *p* = 0.002), and T5 ($\chi ^2(2)$ = 48.00, *p* < 0.001).

Subsequently, we performed post hoc analysis using Wilcoxon signed-rank tests with a Bonferroni correction for multiple comparisons. This statistical analysis was also performed using SPSS (version 26). For the BART model, there were statistically significant differences in RSA scores between all conditions: Full time (*Mdn* = 0.0326) and Early window (*Mdn* = 0.0199) (*p* = 0.002), Full time and Late window (*Mdn* = 0.0226) (*p* = 0.002), and Early and Late window (*p* = 0.005). For the PEGASUS model, post hoc analysis revealed no statistically significant difference in RSA scores between Full time (*Mdn* = 0.0391) and Early window (*Mdn* = 0.0397) (*p* = 0.196). However, there were statistically significant differences between Full time and Late window (*Mdn* = 0.0238) (*p* = 0.013) and between Early and Late window (*p* = 0.001). Finally, for the T5 model, there were statistically significant differences in RSA scores between Full time window (*Mdn* = -0.0024 and Early window (*Mdn* = 0.0024) (*p* < 0.001), between Full time and Late window(*Mdn* = -0.0061) (*p* < 0.001), and between Early and Late window (*p* < 0.001).

The results revealed distinct processing patterns across language models for various spatiotemporal windows of linguistic information. BART exhibited the highest similarity to human brain activity in comprehensive information processing (Full time window), followed by the P600-associated window (Late window), and lastly, the N400-associated window (Early window). This finding possibly indicates that BART excels in capturing overall language processing and potentially syntax-related information. PEGASUS demonstrated similar processing for comprehensive and N400-associated information but diverged significantly in the P600-associated window. This pattern may indicate consistent handling of general and potentially semantic-related information, with a distinct approach to syntax-related information. T5 showed the highest similarity in the N400-associated window, followed by the comprehensive condition, and lowest in the P600-associated window. This finding suggests that T5’s processing patterns align more closely with human brain activity in spatiotemporal windows associated with the N400 component. These disparities likely reflect the architecture, pretraining methods, and task adaptability differences of DLMs. For instance, BART’s sequence-to-sequence pretraining may enhance structural information processing, while PEGASUS’s summarization-focused design may prioritize semantic information. The findings provide insights into how various language models process different components of human language comprehension.

We also employed Wilcoxon signed-rank tests to determine whether significant differences existed between each model’s three experimental conditions and their respective control conditions. The results are presented in Table 3. A positive *Z*-score indicates that the experimental condition surpassed the control condition. At the same time, a *p*-value < 0.05 denotes a statistically significant difference between the two.

**Table 3:** Wilcoxon signed-rank test results for differences between experimental and control conditions across models.

**Comparison pairs**	**BART**		**PEGASUS**		**T5**	
	***Z***	***p***	***Z***	***p***	***Z***	***p***
Full time window—baseline	3.059	0.002**	2.327	0.020*	2.857	0.004**
Full time window—control (rand.init., Full)	2.934	0.003**	3.516	<0.001***	1.229	0.219
Early window—baseline	3.059	0.002**	2.689	0.007**	1.829	0.067
Early window—control (rand.init., Early)	3.059	0.002**	3.464	0.001**	4.286	<0.001***
Late window—baseline	3.059	0.002**	1.813	0.056	3.743	<0.001***
Late window—control (rand.init., Late)	2.981	0.003**	3.516	<0.001***	3.771	<0.001***

*$$p<.05$$*; **$$p<.01$$; ***$$p<.001$$.

The results demonstrate that all experimental conditions for each model exceeded their corresponding control conditions (*Z* > 0), with the majority achieving statistical significance (*p* < 0.05). This finding suggests that trained language models can acquire LRs more akin to those of the human brain and, in most cases, significantly differentiate between language processing states (Full time, Early and Late window) and baseline states (Baseline). These findings underscore the crucial role of model training in attaining brain-like LRs.

The RSA score comparisons that do not reach statistical significance include the PEGASUS model’s Late window vs. Baseline, the T5 model’s Full time window vs. Control (rand.init., Full), and Early window vs. Baseline. As illustrated in Fig. 5, in the shallower layers of each model, the experimental conditions’ RSA scores are lower than those of the control conditions. However, as the layer depth increases, the experimental conditions’ RSA scores consistently surpass those of the control conditions from a particular layer onwards. This phenomenon indicates that as the network deepens, the model progressively learns representations that better distinguish between language processing and baseline states. Baseline, serving as the baseline before stimulus presentation, represents a non-specific language processing state. As the layer depth increases, the models’ similarity to language processing-related conditions (Full time, Early and Late window) increases, while the difference from the baseline condition becomes more apparent, suggesting that the models are learning features similar to human brain language processing. Concurrently, this phenomenon indicates that the effects of training are more pronounced in the deeper layers of the model, emphasizing that more profound layers of network structures play a more critical role in capturing the LRs of the human brain.

Finally, we conducted two additional exploratory analyses. First, we performed an RSA across different encoder layers of the 3 DLMs. Specifically, we calculated the RSA scores between each pair of encoder layers for BART, PEGASUS, and T5 models. We generated three heatmap matrices based on these RSA scores, as illustrated in Supplementary Fig. S1. Supplementary Fig. S1a and Fig. S1c correspond to the inter-layer similarities between BART-PEGASUS, BART-T5, and PEGASUS-T5. We normalized the matrices to a square format to facilitate a comparison between models with different numbers of layers. This visualization approach revealed several interesting trends. For BART and PEGASUS, the RS between hidden layers initially increased with depth but decreased in the deeper layers. The representational similarity between BART and T5 hidden layers showed an increasing trend as the layer depth increased. Conversely, the RS between PEGASUS and T5 hidden layers exhibited a decreasing trend with increasing layer depth. These findings offer valuable insights that could inform future comparative analyses of representational patterns across different models.

We performed two exploratory RSA-based analyses to comprehensively interpret the experimental results.

In the first analysis, we calculated the RSA scores between each pair of encoder layers across the BART, PEGASUS, and T5 models. We visualized these scores as three heatmap matrices in Supplementary Fig. S1, normalizing them to a square format to facilitate comparison between models with different numbers of layers. The visualization revealed extensive regions of similarity (depicted in blue), indicating potential convergence in RPs across these DLMs. Notably, PEGASUS and T5 demonstrate higher inter-model RS than their respective similarities with BART, consistent with the visualization patterns observed in Figure 4. Furthermore, our analysis revealed significant correlations between the deeper hidden layers of all three DLMs and human brain activity patterns. These results support the Platonic Representation Hypothesis^55^, which suggests that, given adequate training data and model capacity, neural networks naturally converge toward a common statistical representation of reality.

In the second analysis, we calculated the correlation between the RSMs obtained under three conditions for each participant and then averaged the results across all participants. The results reveal a Spearman correlation of 0.439 between Full time and Early window, 0.466 between Full time and Late window, and 0.273 between Early and Late window. All 13 participants demonstrate statistically significant correlations (*p* < 0.05) across the three comparisons. These findings indicate high correlations among the RPs of EEG under the three conditions, notably higher than the RSA scores between human brain activity and DLMs. We posit that this outcome is reasonable. Firstly, it relates to the construction method of the EEG vectors. Full time window effectively encompasses all the information from Early and Late window, including time durations and electrode locations. Moreover, Early and Late window partially overlap, sharing specific EEG electrodes and being temporally separated by only 50ms. This inherent interconnection in the data structure may contribute to the high similarity between conditions. Secondly, this analysis compares different RPs within the same neural system (the human brain). In contrast, we compare patterns between two independent systems when we conduct RSA between human brain activity and DLMs. Therefore, it is reasonable that RSA scores across different EEG conditions are higher than those between brain activity and models. This reasoning also applies to the observation that inter-layer RSA scores within DLMs are higher than those between brain activity and DLMs, as all three DLMs are fundamentally based on the Transformer architecture, and we have extracted representations from their encoder layers. Lastly, previous research^41^ analyzing the RS between human EEG signals and model embeddings has reported RSA scores (correlation coefficients) on the order of $10^{-2}$. To some extent, this finding supports the notion that this magnitude of correlation between human brain activity and model representations is indeed meaningful and valid.

### Impact of layer manipulation on model performance

Tables 4, 5 and 6 present the Rouge metrics for three DLMs after conducting layer-wise attention ablation or noise addition, reflecting the models’ performance on the ABS task. The notation “w/o” indicates that no manipulation was applied to any encoder layer, while “all” signifies that all encoder layers were processed. The bolded values represent the Rouge scores that showed the greatest performance decline after attention ablation or noise addition. Perplexity is a control variable for the correlation analysis in the following subsection. In language model evaluation, a lower perplexity score indicates that the model is better at predicting the text.

**Table 4:** BART model performance after layer manipulation.

**Ablated** **layer**	**Rouge1**	**Rouge2**	**RougeL**	**Perplexity**	**Noisy** **layer**	**Rouge1**	**Rouge2**	**RougeL**	**Perplexity**
w/o	0.4570	0.2172	0.4839	6.5545	w/o	0.4570	0.2172	0.4839	6.5545
1	0.4536	0.2172	0.4213	6.9029	1	0.4571	0.2223	0.4249	6.5728
2	0.4476	0.2144	0.4159	7.4548	2	0.4557	0.2219	0.4239	6.6231
3	0.4497	0.2142	0.4171	7.7634	3	0.4545	0.2214	0.4224	6.6872
4	0.4514	0.2178	0.4194	6.8744	4	0.4577	0.2227	0.4256	6.5961
5	0.4443	0.2114	0.4127	7.3242	5	0.4551	0.2205	0.4227	6.7478
6	0.4349	0.2039	0.4041	7.6296	6	0.4507	0.2180	0.4188	6.9318
7	0.4417	0.2107	0.4102	7.2417	7	0.4540	0.2183	0.4218	6.8882
8	0.4301	0.2044	0.3993	7.5065	8	0.4501	0.2158	0.4182	6.9067
9	0.4356	0.2021	0.4039	7.8835	9	0.4507	0.2182	0.4192	6.7484
10	0.4409	0.2071	0.4090	7.2498	10	0.4517	0.2162	0.4207	6.3082
11	0.4337	0.1984	0.4029	6.5815	11	**0.4425**	**0.2106**	**0.4110**	6.4026
12	**0.3840**	**0.1677**	**0.3572**	6.9424	12	0.4491	0.2133	0.4174	5.9276
All	0.0549	0.0024	0.0527	1.1475	all	0.2653	0.0763	0.2444	10.0152

**Table 5:** PEGASUS model performance after layer manipulation.

**Ablated** **layer**	**Rouge1**	**Rouge2**	**RougeL**	**Perplexity**	**Noisy** **layer**	**Rouge1**	**Rouge2**	**RougeL**	**Perplexity**
w/o	0.4835	0.2485	0.3978	4.4455	w/o	0.4835	0.2485	0.3978	4.4455
1	0.4839	0.2482	0.3981	3.8162	1	0.4843	0.2487	0.3982	5.8081
2	0.4837	0.2482	0.3975	4.6588	2	0.4836	0.2484	0.3979	4.5298
3	0.4816	0.2463	0.3943	4.4042	3	0.4833	0.2479	0.3973	4.4933
4	0.4818	0.2462	0.3955	4.6315	4	0.4838	0.2485	0.3976	4.5074
5	0.4827	0.2478	0.3967	4.4057	5	0.4844	0.2488	0.3980	4.4795
6	0.4813	0.2466	0.3957	4.5811	6	0.4834	0.2488	0.3974	4.5506
7	0.4828	0.2481	0.3974	4.8670	7	0.4837	0.2490	0.3984	4.5029
8	0.4816	0.2472	0.3959	4.7064	8	0.4824	0.2477	0.3966	4.5299
9	0.4813	0.2465	0.3957	4.2938	9	0.4817	0.2468	0.3955	4.5912
10	0.4819	0.2469	0.3961	4.7257	10	0.4813	0.2466	0.3956	4.9480
11	**0.4790**	**0.2440**	0.3926	4.6511	11	0.4803	0.2447	0.3942	4.6066
12	0.4811	0.2459	0.3950	4.7428	12	0.4812	0.2460	0.3950	4.5174
13	0.4801	0.2448	0.3938	4.7486	13	0.4793	0.2449	0.3934	4.4523
14	0.4794	0.2442	**0.3926**	4.5026	14	0.4797	0.2448	0.3938	4.4388
15	0.4809	0.2461	0.3940	4.5126	15	0.4788	0.2433	0.3916	4.4305
16	0.4801	0.2452	0.3935	4.6827	16	**0.4774**	**0.2419**	**0.3910**	4.5789
All	0.1790	0.0275	0.1351	4.2754	all	0.4364	0.2011	0.3459	6.5883

**Table 6:** T5 model performance after layer manipulation.

**Ablated** **layer**	**Rouge1**	**Rouge2**	**RougeL**	**Perplexity**	**Noisy** **layer**	**Rouge1**	**Rouge2**	**RougeL**	**Perplexity**
w/o	0.3600	0.1432	0.2881	4.2509	w/o	0.3600	0.1432	0.2881	4.2509
1	0.3579	0.1443	0.2892	4.4911	1	0.3598	0.1428	0.2880	5.2915
2	0.3620	0.1442	0.2901	6.5301	2	0.3596	0.1425	0.2879	4.6982
3	0.3586	0.1415	0.2865	4.9183	3	0.3597	0.1424	0.2877	4.8546
4	0.3575	0.1408	0.2859	5.0020	4	0.3591	0.1425	0.2874	4.7872
5	0.3605	0.1432	0.2887	4.8580	5	0.3589	0.1423	0.2873	4.7402
6	0.3596	0.1425	0.2877	4.7825	6	0.3588	0.1423	0.2871	4.9763
7	0.3591	0.1423	0.2875	4.9920	7	0.3596	0.1429	0.2883	4.8143
8	0.3583	0.1422	0.2870	4.6951	8	0.3581	0.1423	0.2871	4.7670
9	0.3624	0.1453	0.2909	4.5493	9	0.3597	0.1427	0.2878	4.7384
10	0.3603	0.1434	0.2889	4.5590	10	0.3581	0.1418	0.2865	4.9308
11	0.3600	0.1430	0.2880	4.8998	11	0.3596	0.1430	0.2879	4.8856
12	0.3607	0.1434	0.2886	5.1357	12	0.3589	0.1427	0.2875	4.6917
13	0.3582	0.1413	0.2865	4.7659	13	0.3575	0.1413	0.2864	4.9292
14	0.3590	0.1425	0.2877	4.9346	14	0.3574	0.1414	0.2862	4.7829
15	0.3574	0.1414	0.2859	5.0197	15	0.3587	0.1424	0.2869	5.0375
16	0.3567	0.1401	0.2853	5.0489	16	**0.3571**	0.1413	0.2860	4.8089
17	0.3553	0.1398	0.2842	5.1154	17	0.3580	0.1415	0.2864	4.8560
18	0.3568	0.1408	0.2852	5.5020	18	0.3582	0.1417	0.2864	5.1116
19	0.3536	0.1385	0.2823	5.4765	19	0.3579	0.1412	0.2866	4.9381
20	**0.3535**	**0.1379**	**0.2818**	5.2662	20	0.3595	0.1423	0.2878	4.9578
21	0.3549	0.1392	0.2837	4.5478	21	0.3581	0.1418	0.2866	4.5155
22	0.3556	0.1394	0.2842	4.3239	22	0.3565	**0.1405**	**0.2851**	5.3488
23	0.3551	0.1387	0.2836	4.9647	23	0.3595	0.1427	0.2877	4.6696
24	0.3581	0.1395	0.2864	5.6963	24	0.3593	0.1423	0.2876	4.9935
All	0.1427	0.0139	0.1111	3.7422	all	0.3396	0.1294	0.2717	5.0026

**Figure 6:**
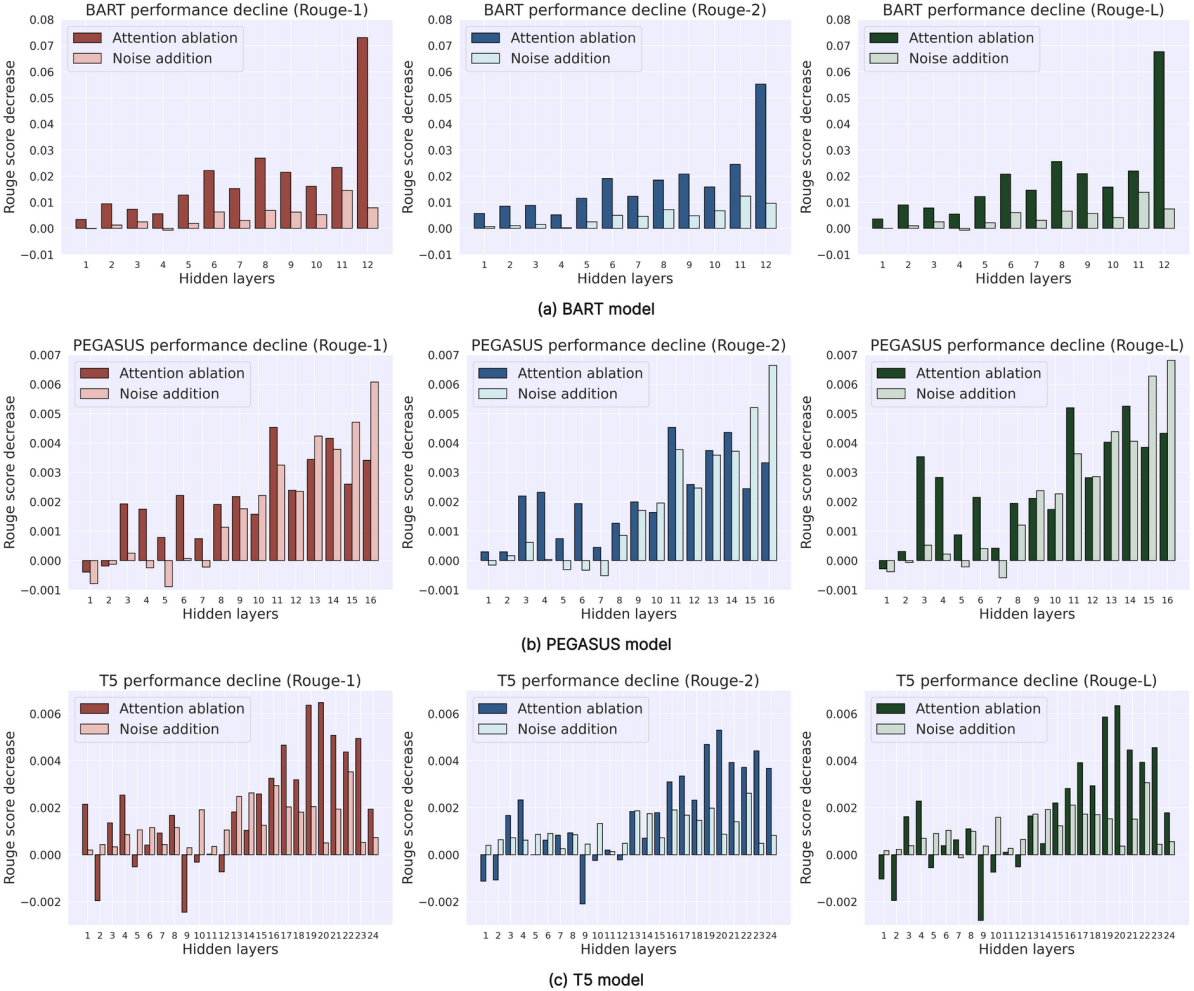
Impact of layer-wise attention ablation and noise addition on ABS task performance.

Figure 6 graphically demonstrates the impact of ablating attention and noise addition at each layer on the ABS task, with negative numbers indicating performance improvements. This subsection primarily describes experimental results to lay the groundwork for analysis in the following subsection. For BART, attention ablation in any encoder layer decreases all Rouge metrics, most significantly in the final layer. Noise introduction causes the most significant decline at layer 11. Interestingly, layer 4 shows slight increases in Rouge1 and RougeL. From layer 3 onwards, attention ablation degrades performance in PEGASUS, with deeper layers having a more significant impact. The 11th layer’s ablation most significantly reduces Rouge1 and Rouge2. The 14th layer’s uniform attention distribution causes the most significant RougeL decrease. Noise introduction from layer 8 onwards reduces all Rouge metrics, peaking at the final layer. For T5, performance degradation due to attention ablation begins at layer 13. Earlier layers show performance fluctuations, with some improving all Rouge metrics. From layers 13 to 24, performance consistently declines, maximally at layer 20. Noise introduction generally decreases all Rouge metrics (except RougeL in the lower seven layers), with peak drops at layers 16 (Rouge1) and 22 (Rouge2 and RougeL).

Notably, the changes in Rouges for the PEGASUS and T5 models are considerably more minor than those observed in the BART model, suggesting that the increased number of layers in PEGASUS and T5 enhances performance stability. However, this does not imply that more layers invariably leads to better performance. For instance, after setting the attention of layer 9 in the T5 model to a uniform distribution, a considerable performance improvement was observed. This suggests that the attention mechanism in this layer may have previously interfered with the effective extraction and integration of crucial information, indicating that not every hidden layer positively contributes to the task of ABS.

### Correlation between RS and model performance

**Figure 7:**
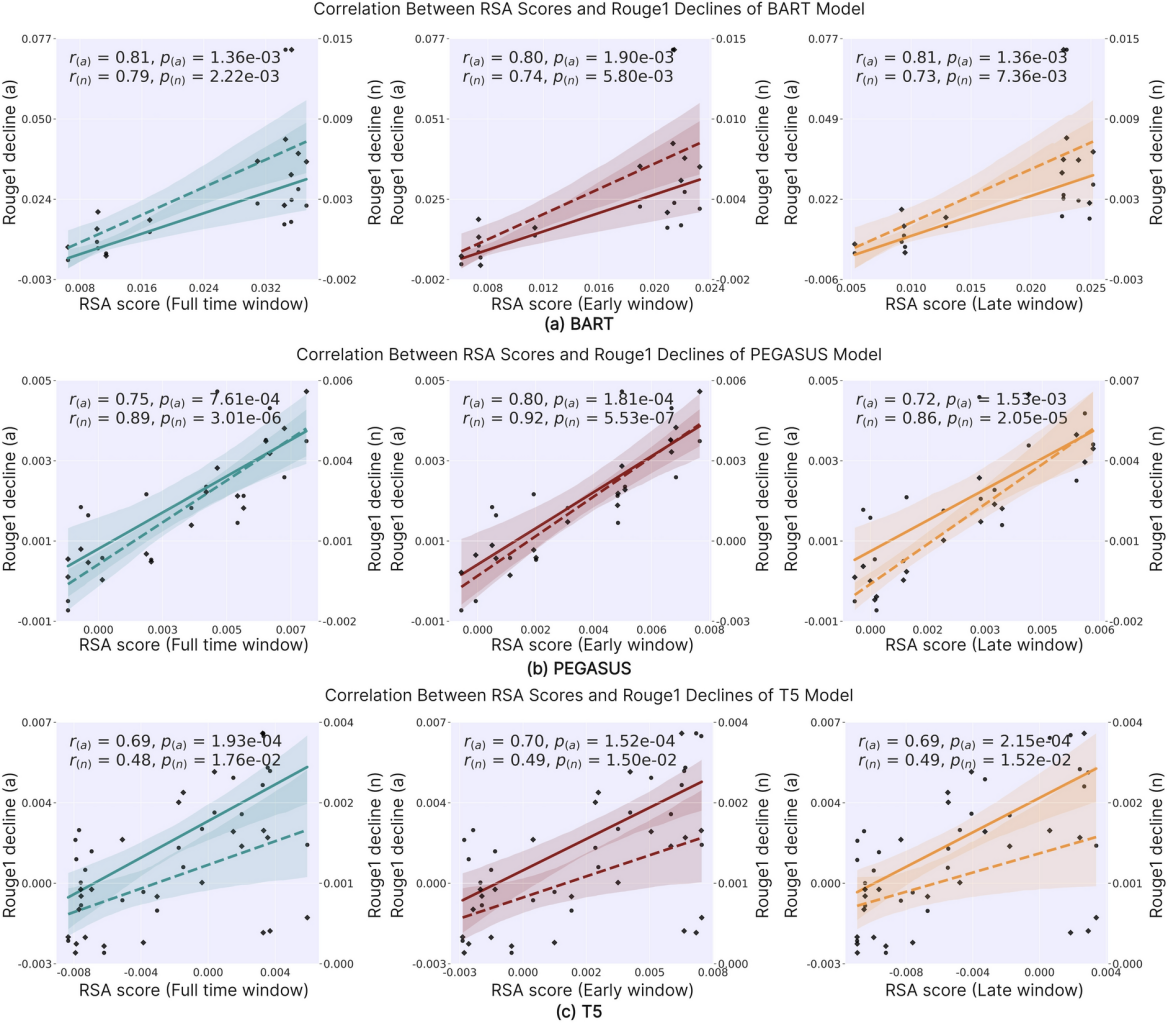
Correlation between RSA scores and Rouge1 declines in BART, PEGASUS, and T5 models.

This subsection presents the experimental results based on a correlation analysis conducted to test the research hypothesis using data from the previous two subsections. Figure 7 and Supplementary Fig. S2 and Fig. S3 illustrate the correlation between model performance (Rouge1/2/L) decline and the RSA scores of each encoder hidden layer for the three ABS models. Each subplot represents the results of correlation analysis under a specific experimental condition of RSA. Specifically, each subplot contains two sets of regression lines and corresponding scatter plots: solid lines with solid scatter points represent the effects of attention ablation (setting each layer’s attention to a uniform distribution). In contrast, dashed lines with diamond scatter points depict the effects of adding noise to each layer. Both sets of regression lines share the same x-axis (RSA scores) but utilize different y-axis. The left y-axis corresponds to the former, representing the performance decline due to attention ablation (Rouge1/2/L decline (a)). The right y-axis corresponds to the latter, indicating the performance decline from noise addition (Rouge1/2/L decline (n)). For example, with its 12 encoder layers, the BART model is represented by 24 data points: 12 circle points for attention ablation and 12 diamond points for noise addition, each corresponding to a specific layer. The shaded areas surrounding each regression line represent the 95% confidence intervals (95%*CI*). the Spearman correlation coefficients presented in this subsection were calculated using Python 3.7.9. The ‘*r*’ value in the figure represents the Spearman correlation coefficient, and ‘*p*’ denotes the significance level of the correlation, with a *p*-value < 0.05 signifying a statistically significant correlation. To better focus on the main findings of our study, we have opted to include only the figures and tables related to Rouge1 in the main text. The Supplementary Fig. S2 and Fig. S3 and tables showing results for Rouge2 and RougeL can be found in the Appendix.

Figure 7 demonstrates that for the three DLMs, the RSA scores calculated under three conditions show a significant positive correlation (*p* < 0.05) with the model performance decline (Rouge1) caused by two types of encoder layer operations. Similar results are observed for Rouge2 and RougeL in Supplementary Fig. S2 and Fig. S3. In other words, hidden layers with LRs more closely aligned to the human brain exhibit more pronounced model performance deterioration when manipulated. This finding supports our hypothesis that the hidden layers of the model that exhibit a higher degree of RS to the human brain’s activation patterns may play a more critical role in the model’s performance on the ABS task.

To enhance the preciseness of our findings, we used approximate non-parametric partial correlation analysis to control for perplexity’s influence. We first calculated Spearman correlations among Rouge, RSA, and perplexity. To remove perplexity’s impact, we computed partial correlations between Rouge and RSA using linear regression to estimate perplexity’s effect, then calculated residuals. Finally, we determined Spearman correlations between these residuals, yielding partial correlation coefficients. This method excluded potential confounding effects from perplexity, allowing for a more accurate assessment of relationships between variables.

Table 7 presents the results of partial correlation analysis for Rouge1 scores. The study reveals that, even when controlling for perplexity, the RSA scores maintain a significant positive correlation (*p* < 0.05) with the performance decline caused by two types of encoder layer operations across all three experimental conditions for the three DLMs. Supplementary Table S2 and Table S3 show essentially consistent results for Rouge2 and RougeL, except the positive correlation between RSA scores and Rouge2 decline for the T5 model in Full time window narrowly missed statistical significance (*p* = 0.051). These findings unveil a deep structure-function relationship that transcends the models’ general language understanding capabilities while emphasizing the crucial role of specific neural network layers in the ABS task. These layers may perform functions analogous to the human brain in language compression and key information extraction processes.

**Table 7:** Partial correlation analysis of RSA scores and Rouge1 performance decline.

Conditions	Full time window		Early window		Late window	
	*r’*	*p’*	*r’*	*p’*	*r’*	*p’*
BART(a)	0.804	0.002**	0.790	0.002**	0.734	0.007**
BART(n)	0.790	0.002**	0.776	0.003**	0.776	0.003**
PEGASUS(a)	0.900	<0.001***	0.935	<0.001***	0.897	<0.001***
PEGASUS(n)	0.744	<0.001***	0.788	<0.001***	0.718	0.002**
T5(a)	0.723	<0.001***	0.737	<0.001***	0.722	<0.001***
T5(n)	0.434	0.034*	0.454	0.026*	0.447	0.029*

Notably, this correlation persists across different model architectures (BART, PEGASUS, T5), various conditions of human brain LR (Full time, Early and Late window), different perturbation methods (attention ablation, noise injection), and multiple performance metrics (Rouge1, 2, L). This consistency and robustness suggest the possible existence of universal neural computational principles independent of specific model implementations.

To comprehensively evaluate model performance, we invite five native speakers to manually score summaries generated by BART and PEGASUS models. We randomly select ten articles from the test set to assess the model summaries after attention ablation. The evaluation metrics include informativeness, readability, and factual consistency, with scores ranging from 1 to 5, where higher scores indicate better performance. Detailed evaluation criteria are presented in Supplementary Table S4. We do not evaluate the T5 model, as it generates 25 summaries per article, which could lead to evaluator fatigue and introduce errors. We calculate the correlation between the three metrics of model summaries and the RSA scores of affected hidden layers and the human brain, with results shown in Table 8 (BART) and Table 9 (PEGASUS). For BART, the three metrics show negative correlations with RSA scores under all three conditions, although these correlations do not reach statistical significance. This result suggests that model performance tends to decline when hidden layers more similar to human brain representations are disrupted. PEGASUS exhibits identical trends in Full time and Early window, while in Late window, RSA scores are only moderately negatively correlated with readability.

**Table 8:** Correlation Between the RSA Scores and Manual Ratings of BART Model.

Conditions	Informativeness		Readability		Factual consistency	
	*r*	*p*	*r*	*p*	*r*	*p*
Overall	-0.455	0.137	-0.336	0.285	-0.343	0.276
N400	-0.438	0.155	-0.336	0.285	-0.336	0.286
P600	-0.328	0.301	-0.235	0.463	-0.266	0.404

**Table 9:** Correlation Between the RSA Scores and Manual Ratings of PEGASUS Model.

Conditions	Informativeness		Readability		Factual Consistency	
	*r*	*p*	*r*	*p*	*r*	*p*
Overall	-0.112	0.679	-0.308	0.245	-0.091	0.736
N400	-0.131	0.628	-0.358	0.173	-0.109	0.687
P600	0.003	0.991	-0.279	0.295	0.016	0.952

Overall, the manual evaluation results align with the trends observed in Rouge evaluations (Rouge uses decreased values in evaluation, hence the positive correlation). Although the correlations in the manual assessment do not reach significant levels, they also support our hypothesis: hidden layers with representations similar to human brain language patterns play a crucial role in summarization tasks.

## Discussion

This study proposes a central hypothesis: the hidden layers of a Deep Language Model (DLM) that exhibit a higher degree of Representational Similarity (RS) to the human brain’s activation patterns may play a more critical role in the model’s performance. We employed EEG experiments and Representational Similarity Analysis (RSA) to systematically investigate the similarity between the Language Representations (LRs) of hidden layers in Abstractive Summarization (ABS) models and human language understanding processes to validate this hypothesis. We further explored the correlation between this brain-model similarity and model performance through layer-wise attention ablation and noise addition. The experimental results consistently supported our hypothesis across diverse testing conditions, demonstrating robust evidence for our claim.

Through the RSA of three representative ABS models, we derived several findings that hold significant implications for understanding the internal LR mechanisms of DLMs and their relationship to human language cognition. We observed a notable trend: as the model layers deepened, the similarity between encoder layers and human brain language processing generally showed an upward trend, reaching statistical significance (*p* < 0.05) under most experimental conditions. However, this finding differs from some previous studies. For instance, research^56^ found that the 9th layer (out of 12) of GPT-2 was most similar to human brain activity. At the same time^10^, it was discovered that the middle layers (6 or 7 out of 12) of the BERT model showed the highest similarity to human brain activity. This apparent discrepancy reflects several issues worthy of in-depth exploration. Firstly, although our experimental results show a statistically significant monotonic increase in RSA scores between the model and the human brain as layer depth increases, this does not preclude the possibility of local fluctuations in intermediate hidden layers. For example, under Full time window, the BART model showed a temporary decrease starting from the 6th layer, and the difference in RSA scores between the 6th layer and the 12th (final) layer was insignificant (*p* = 0.385). Focusing on individual layer comparisons rather than the overall trend might lead to different conclusions. Secondly, our study employed an encoder-decoder model fine-tuned on the ABS task to analogize the complete process of human language understanding and generation. In contrast, GPT-2 and BERT use decoder-only and encoder-only architectures, respectively. This task-specific architectural difference may result in different representation learning patterns. Moreover, the ABS task demands more robust semantic integration and information compression capabilities from the model, potentially driving deeper networks to form high-level LRs more akin to the human brain. Lastly, control experiments using randomly initialized models indicate that the pre-training and fine-tuning processes play a crucial role in shaping the model’s representational characteristics. This finding underscores the importance of task-specific training in molding internal model representations. Thus, specific fine-tuning for the ABS task may alter the LRs of DLMs, causing deeper networks to focus more on language feature extraction relevant to the ABS task. Our findings help to reveal the complex interplay between DLM architecture, task-specific training, and models’ similarity to human brain language processing. This study underscores the importance of considering these factors when comparing DLMs to human cognition, providing a foundation for future research in developing more human-like language models.

The core findings of this study reveal that hidden layers in DLMs with higher similarity to human brain LRs have a more significant impact on model performance. This correlation demonstrates remarkable robustness across multiple dimensions: various DLM types (BART, PEGASUS, T5), different human brain LR conditions (Full time, Early and Late window), diverse layer manipulation methods (attention ablation, noise addition), and persists even after controlling for perplexity. The consistency and robustness of these findings warrant in-depth discussion from multiple perspectives. From the architectural standpoint of DLMs, although BART, PEGASUS, and T5 models differ in the number of hidden layers, training objectives, and fine-tuning datasets, they all share the encoder-decoder Transformer architecture as a common foundation. The shared underlying structure may preserve similar information processing mechanisms and representation learning patterns, potentially as a critical factor in the observed cross-model similarities. Considering the human brain LR conditions, while Full time, Early and Late window focus on different time windows and electrode locations, they likely capture partially overlapping language processing information. This spatiotemporal overlap in neural signals may contribute to the consistency across conditions. Moreover, DLMs may simultaneously learn LRs corresponding to multiple stages of brain processing, enabling them to exhibit similarities with human brain patterns across different conditions. From the perspective of layer-wise operations, layers with LRs more closely aligned to human brain language patterns may perform specific functions crucial to the ABS task, such as high-level semantic integration or contextual understanding. Consequently, whether through attention ablation or noise addition, directly impacting the model’s ability to process critical linguistic information leads to significant performance degradation. Regarding the control variable, perplexity primarily reflects a model’s general language modeling capability. However, the ABS task requires not only basic language comprehension but also the ability to extract and compress critical information. LRs in DLMs similar to those in the human brain may excel at this advanced information processing and abstraction, which is not fully captured by the perplexity metric. These findings highlight a crucial link between the neural representations in DLMs and human brain language processing, suggesting that the brain-like layers are also critical for high-level language tasks. This convergence, robust across model architectures and experimental conditions, implies that DLMs may be capturing fundamental aspects of human language processing.

Throughout our research process, we have identified several limitations in the current study, which provide directions for future research. Firstly, this study did not conduct an in-depth comparative analysis of the experimental results among the three DLMs. Our research design selected BART, PEGASUS, and T5 models, which vary in type, hidden layer count, and fine-tuning dataset, to enhance the consistency and robustness of our hypotheses across diverse model types. However, this approach limited our ability to isolate the effects of individual factors. Future research could adopt more rigorous variable control strategies to investigate these factors independently. For example, comparing BART and PEGASUS models with identical layer counts and fine-tuning datasets could isolate the influence of model type. Similarly, examining T5 variants that differ only in parameter count while keeping other conditions constant could focus on the impact of model scale on performance. These targeted comparisons would provide more nuanced insights into the specific contributions of each factor to the observed effects. Secondly, this study primarily focused on the overall trends of RSA scores across different experimental conditions without delving into changes at the individual hidden layer level. Therefore, future experiments could be designed to cross-validate RSA scores with findings from other interpretability research methods, such as probing tasks or feature visualization, to understand the model’s internal representations better. Furthermore, although we set three EEG signal conditions, this approach still faces challenges separating semantic and syntactic representations within EEG signals. However, studies^18,19,56^ have, in turn, provided approaches for separating syntactic and semantic representations in GPT-2 (decoder-only). Future research could attempt to adapt and transfer this method to encoder architectures, enabling separation and analysis of syntactic and semantic representations within encoder layers. Lastly, the recent emergence of decoder-only open-source Large Language Models (LLMs), such as LLaMA^57^, Vicuna^58^, and Mistral^59^, has captured significant attention from academia and industry. Despite lacking explicit encoder structures, these models demonstrate remarkable language understanding capabilities and show potential for application in ABS tasks. Future research could explore the relationships between these LLMs and human language cognition. Specifically, comparative analyses could focus on aspects such as parameter scale, pre-training strategies, and performance on downstream tasks. Such investigations could provide valuable insights into the similarities and differences between these advanced AI models and human language processing, potentially bridging the gap between artificial and biological approaches to language understanding.

## Conclusion

This study investigated the similarity between the deep language models’ encoder hidden layer representations and human brain activity during language processing using EEG-based experiments and representational similarity analysis. Subsequently, we employed a hierarchical manipulation approach to analyze the correlation between this brain-model similarity and model performance. Our core hypothesis was verified: the hidden layers of a model that exhibit a higher degree of representational similarity to the human brain’s activation patterns play a more critical role in the model’s performance on the abstractive summarization task. This correlation manifested and attained statistical significance across various experimental conditions. These conditions encompassed diverse deep language model types (BART, PEGASUS, T5), multiple spatiotemporal windows representing distinct phases of human language representation, various hierarchical manipulation techniques including attention ablation and noise addition, and an array of model performance evaluation metrics (Rouge1, Rouge2, RougeL). Notably, this correlation persisted even after accounting for the confounding influence of perplexity on model performance. The heterogeneity of these experimental conditions bolsters the robustness and validity of our conclusions, yielding profound insights into the alignment between deep language models and human language processing mechanisms.

## Supplementary Information

Supplementary Information.

## Data Availability

The EEG data and model hidden states are available at https://drive.google.com/drive/folders/1-C_e_HqdS6MIf1gGVehG7DW6B0MQguco?usp=sharing.

## Code availability

The code for RSA (with data), layer manipulation, and correlation computation (with data) are also available at https://drive.google.com/drive/folders/1-C_e_HqdS6MIf1gGVehG7DW6B0MQguco?usp=sharing.
