# Supplementary material for: Brain-model neural similarity reveals abstractive summarization performance
Source: Sci Rep. 2025 Jan 2;15:370. doi: 10.1038/s41598-024-84530-w (PMC11696092; doi:10.1038/s41598-024-84530-w)
Supplement: Supplementary file 1 — Supplementary Information. [file 41598_2024_84530_MOESM1_ESM.pdf]

Supplementary Information

Supplementary Table S1. Details of experimental materials

Article	Count of sentences	Count of words
1	9	207
2	10	180
3	11	242
4	7	176
5	6	148
6	6	143
7	7	160
8	6	171
Total	62	1427

Supplementary Table S2. Partial Correlation Analysis of RSA Scores and Rouge2 Performance Decline

Conditions	Full time window		Early window		Late window	
	r'	*p'*	*r'*	*p'*	*r'*	*p'*
BART(a)	0.741	0.006**	0.748	0.005**	0.629	0.028*
BART(n)	0.776	0.006**	0.755	0.005**	0.811	0.001**
PEGASUS(a)	0.691	0.003**	0.756	< 0.001***	0.685	< 0.003**
PEGASUS(n)	0.856	< 0.001***	0.885	< 0.001***	0.824	< 0.001***
T5(a)	0.739	< 0.001***	0.768	< 0.001***	0.762	< 0.001***
T5(n)	0.403	0.051	0.411	0.046*	0.407	0.048*

Supplementary Table S3. Partial Correlation Analysis of RSA Scores and RougeL Performance Decline

Conditions	Full time window		Early window		Late window	
	r'	*p'*	*r'*	*p'*	*r'*	*p'*
BART(a)	0.804	0.002**	0.790	0.002**	0.734	0.007**
BART(n)	0.790	0.002**	0.776	0.003**	0.776	0.003**
PEGASUS(a)	0.629	0.009**	0.688	0.003**	0.618	0.011*
PEGASUS(n)	0.891	< 0.001***	0.932	< 0.001***	0.894	< 0.001***
T5(a)	0.703	< 0.001***	0.753	< 0.001***	0.741	< 0.001***
T5(n)	0.420	0.041*	0.438	0.032*	0.430	0.0357*

Supplementary Table S4. Criteria for Human Evaluation of Summaries

Score	The summary
**Informativeness**	
1	Is entirely unrelated to the original text.
2	Has a weak association with the original text, capturing only some minor points.
3	Has a certain degree of relevance to the original text, capturing some key points.
4	Closely relates to the original text capturing critical points.
5	Fully captures the core of the original text and is consistent with the reference summary.
**Readability**	
1	Is difficult to understand, with frequent grammatical errors and logical confusion.
2	Has poor readability, with numerous grammatical and logical issues.
3	Has average readability, with some grammatical or logical problems.
4	Has good readability, with only a few grammatical or logical issues.
5	Has excellent readability, with fluent grammar and precise logic.
**Factual Consistency**	
1	Contains many factual errors and is severely inconsistent with the original text.
2	Contains many factual errors and deviates substantially from the original text
3	Contains some factual errors and deviates some what from the original text.
4	Has almost no factual errors and is consistent with the original text.
5	Is utterly free of factual errors and is entirely consistent with the original text.

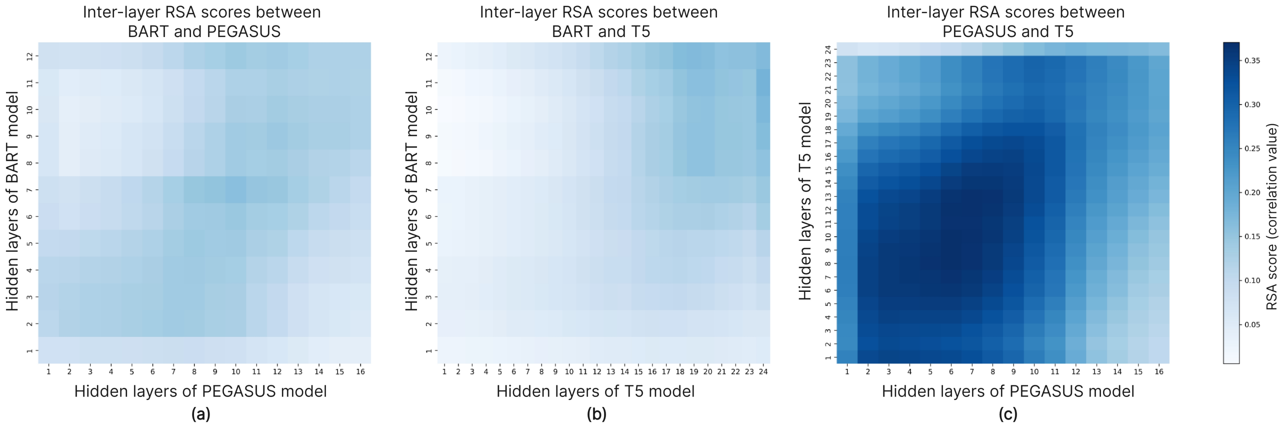

Supplementary Figure S1. Heatmap Matrices of Inter-model Layer-wise RSA

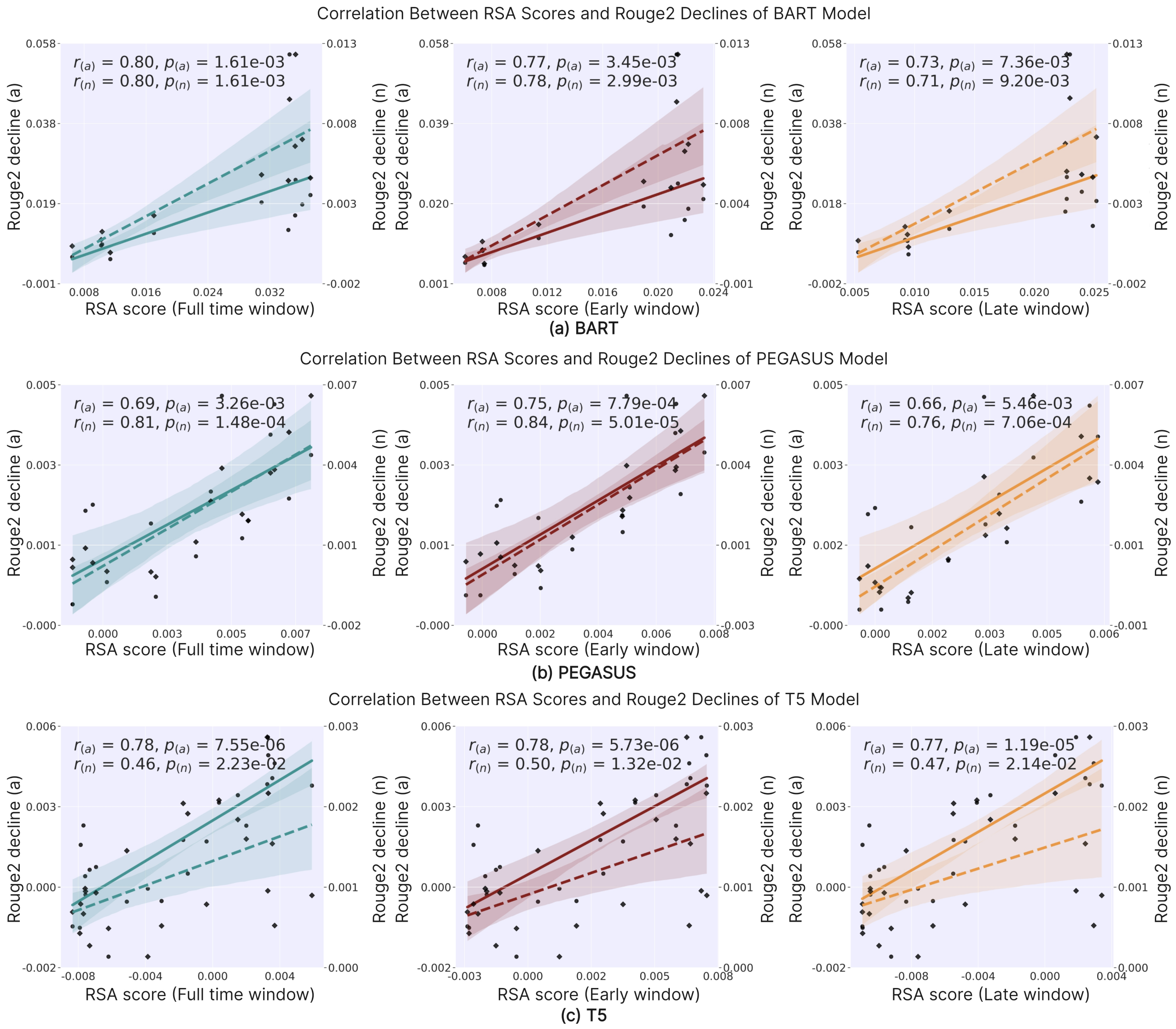

Supplementary Figure S2. Correlation Between RSA Scores and Rouge2 Declines in BART, PEGASUS, and T5 Models

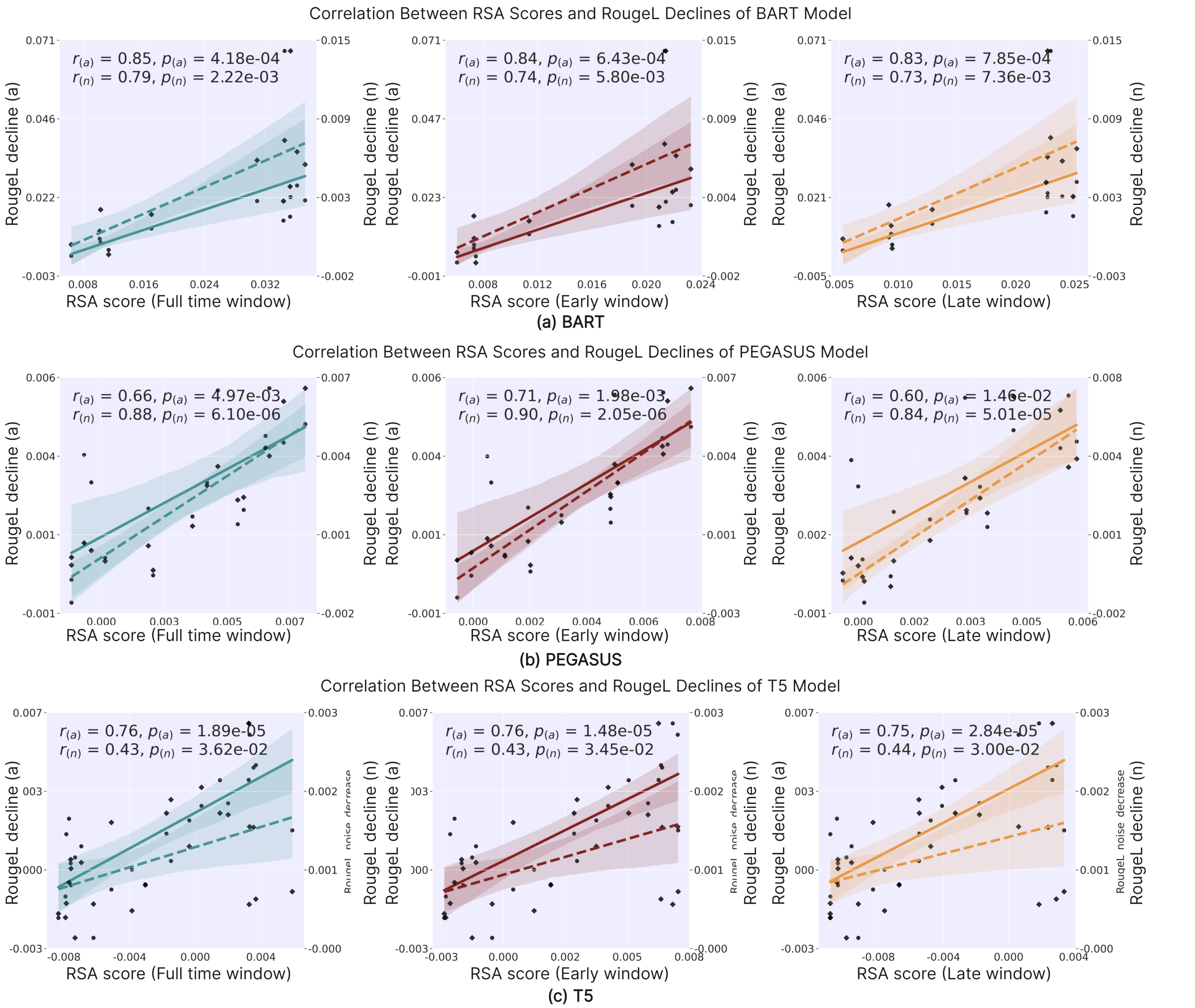

Supplementary Figure S3. Correlation Between RSA Scores and RougeL Declines in BART, PEGASUS, and T5 Models
